# Comparative analysis of radiological and serological methods for diagnosis of cystic echinococcosis

**DOI:** 10.1590/S1984-29612025073

**Published:** 2025-12-12

**Authors:** Baris Can, Busra Betul Ozmen Capin, Meltem Kursun, Canan Cimsit, Aysegul Karahasan Yagci

**Affiliations:** 1 Marmara University Pendik Research and Training Hospital, Department of Medical Microbiology, Istanbul, Turkey; 2 Marmara University Pendik Research and Training Hospital, Department of Radiology, Istanbul, Turkey

**Keywords:** Cystic echinococcosis, CLIA, serology, radiology, Equinococose cística, CLIA, sorologia, radiologia

## Abstract

Cystic echinococcosis (CE) diagnosis relies on clinical signs, serological tests, radiological findings and histopathology. Lack of pathognomonic features or clinician inexperience can make CE diagnosis challenging. In this study, we aimed to investigate the demographic, serological, and radiological characteristics of individuals with a preliminary diagnosis of CE. A total of 1,410 serum samples from 1,265 patients were analysed for the presence of IgG antibodies Anti-*Echinococcus granulosus* IgG using chemiluminescence immunoassay (CLIA) and indirect haemagglutination assay (IHA). Among these, 148 samples showed serological and radiological findings compatible with CE. Echinococcal cysts were confirmed by ultrasonography, computed tomography, or magnetic resonance imaging. Among the 148 patients compatible with CE, 88 were women (59.5%) with a mean age of 45.0 ± 19.1 years, and 60 were men with a mean age of 35.4 ± 18.3 years. Although there was no significant difference between the sexes regarding the number, location, size, or complications, the mean age of female patients was higher than that of male patients. Our findings suggest that radiological methods are essential for assessing cyst size and activity, while serological methods provide complementary diagnostic value and contribute to generating important epidemiological data. As CE is a zoonosis, collaboration with veterinary surveillance is essential for effective control.

## Resumo

O diagnóstico da equinococose cística (EC) baseia-se em sinais clínicos, testes sorológicos, achados radiológicos e histopatologia. A ausência de características patognomônicas e a inexperiência clínica podem dificultar o diagnóstico, sobretudo na prática clínica diária e em serviços com menor experiência. Neste estudo, investigamos características demográficas, sorológicas e radiológicas de indivíduos com diagnóstico preliminar de EC. Foram analisadas 1.410 amostras de soro de 1.265 pacientes para detecção de anticorpos IgG anti-*Echinococcus granulosus* por meio de imunoensaio por quimioluminescência (CLIA) e teste de hemaglutinação indireta (IHA). Destas, 148 apresentaram resultados sorológicos e radiológicos compatíveis com EC. Cistos equinocócicos foram confirmados por meio de ultrassonografia, tomografia computadorizada ou ressonância magnética. Entre os 148 pacientes compatíveis, 88 eram mulheres (59.5%) com média de 45,0±19,1 anos, e 60 eram homens com média de 35,4±18,3 anos. Não houve diferença significativa entre os sexos quanto ao número, localização, tamanho ou complicações; porém, a média de idade das mulheres foi maior. Nossos achados indicam que métodos radiológicos são essenciais para avaliar o tamanho e a atividade dos cistos, enquanto métodos sorológicos oferecem valor diagnóstico complementar e geram dados epidemiológicos relevantes. Como a EC é uma zoonose, a colaboração com a vigilância veterinária é fundamental para um controle eficaz em saúde pública e clínica.

## Introduction

Cystic echinococcosis (CE), listed among the World Health Organization's 20 neglected tropical diseases, is a global zoonotic infection caused by the larval stage of the cestode *Echinococcus granulosus* sensu lato (s.l.) (Casulli, 2021). While CE predominantly impacts the liver and lungs, it can involve other organs or tissues, such as the spleen, brain, and bones. The transmission occurs through the shedding of eggs by definitive hosts, primarily dogs, to intermediate hosts such as livestock, especially sheep. Humans act as dead-end intermediate hosts for *E. granulosus* s.l. Underreporting and misreporting of CE cases contribute to the neglect of this preventable zoonotic infection, leading to severe consequences (Casulli, 2020).

Endemic regions include the Eastern Mediterranean region, Northern Africa, Southern and Eastern Europe, the southern tip of South America, Central Asia, Siberia, and western China (WHO, 2017). In these areas, the incidence of human infection can surpass 30/100,000 person-years, with prevalence rates reaching 5–10% in parts of South America, Central Asia, China, and Africa. Turkey, where CE is endemic, has a prevalence ranging from 0.15% to 1.05% (Ok et al., 2020). A population study in Turkey reported a 0.61% prevalence of abdominal CE (53 patients among 8618 individuals from six provinces), without significant sex differences (Tamarozzi et al., 2018).

The diagnosis of CE relies on clinical signs, radiological findings, serological tests, and examination of cyst fluid for protoscoleces, along with histological findings (Brunetti et al., 2010). A lack of pathognomonic radiological features or clinician experience can make CE diagnosis challenging, with a broad spectrum of differential diagnoses from simple cysts to malignancies (Tamarozzi et al., 2021a). Hydatid cyst serological tests (IHAs, ELISAs, LAs) using parasite fluid antigens show high sensitivity. ELISAs, immunochromatographic tests (ICTs), and Western blots (WBs) reach approximately 90–96% sensitivity for detecting liver cysts, whereas IHAs have lower performance; however, the sensitivity of this method for detecting lung cysts is moderate (approximately 50–65%), and multiple organ involvement yields ≥90% sensitivity (Tamarozzi et al., 2021b; Caushi et al., 2025). Treatment options for liver CE include surgery, medical treatment, percutaneous treatment, and a Watch-and-wait approach, depending on the WHO classification (Kern et al., 2017).

In veterinary medicine, CE has significant economic impacts on livestock production because of organ condemnation at slaughterhouses, decreased productivity, and increased veterinary public health expenditures. The close human–animal–environment interface emphasizes the importance of a One Health approach in the management and prevention of hydatid disease.

The aim of this study was to analyse the demographic features of seropositive CE patients in our hospital and assess the diagnostic power of radiology and serology for patient management.

Rather than a population-based screening, our work specifically analyzes serum samples obtained from patients clinically suspected of CE during the period January 2018–December 2020. This study contributes additional data on CE in Turkey by retrospectively evaluating a relatively large patient cohort using radiological and serological methods in parallel. This combined approach may help to better understand diagnostic performance and epidemiological characteristics in an endemic setting.

## Materials and Methods

### Study settings

This retrospective study was conducted at Marmara University, Pendik Training and Research Hospital, a 550-bed tertiary referral center located in Istanbul, Turkey. Serum samples were collected between January 2018 and December 2020. Only samples obtained from patients with a clinical suspicion of CE were included in the analysis. Cases submitted for unrelated reasons or without suspicion of CE were excluded. Thus, asymptomatic individuals or samples submitted for unrelated pathologies were excluded, reducing the potential bias arising from concurrent conditions.

Some patients contributed more than one serum sample during follow-up, which explains why the total number of serum samples exceeded the number of individual patients. However, for the present analysis only one record per patient was included, and the 148 patients studied represent unique cases without repetition.

### Serological tests

Serum samples were analysed using a chemiluminescence immunoassay (CLIA) (HYDATIDOSIS VIRCLIA® IgG MONOTEST, Vircell) on the VIRCLIA® system (Vircell, Granada, Spain), and the results were processed using dedicated software. The cut-off for a positive result was set at ≥ 1.10 index. Positive sera were subsequently subjected to the Echinococcosis indirect haemagglutination test (IHA; Hydatidose, Fumouze Laboratoires, France) with a cut-off for a positive test of 1:320 titres. Although IHA has lower specificity compared with CLIA, it was used as part of our institutional diagnostic protocol to confirm positive results. This dual-testing approach increases diagnostic reliability in our setting.

### Imaging data

Echinococcal cysts were primarily classified based on ultrasonography (US), computed tomography (CT), and magnetic resonance imaging (MRI) according to the World Health Organization – Informal Working Group on Echinococcosis (WHO-IWGE) classification (WHO-IWGE, 2003). US was the primary imaging modality for the diagnosis of abdominal CE, while CT and MRI were performed in selected cases, particularly for extrahepatic or complicated cysts. Cysts were categorized as active (CE1, CE2, CE3a, or CE3b) or inactive (CE4 or CE5). In patients with ≥ 2 CE cysts, patients were grouped according to the active cyst. If multiple cysts of the same stage were present, the patients were categorized based on the cyst with the largest diameter. Patients with positive serological results but negative radiological findings were excluded from the study to ensure diagnostic consistency.

### Statistical analysis

The data were analysed using SPSS Statistics for Windows, version 23.0 (IBM Corp., Armonk, NY, USA). Descriptive data were compared using chi-square and Mann‒Whitney U tests, whereas differences between groups were assessed using the independent samples t test. A p value < 0.05 was considered statistically significant.

### Diagnostic flowchart

**Figure 1:**
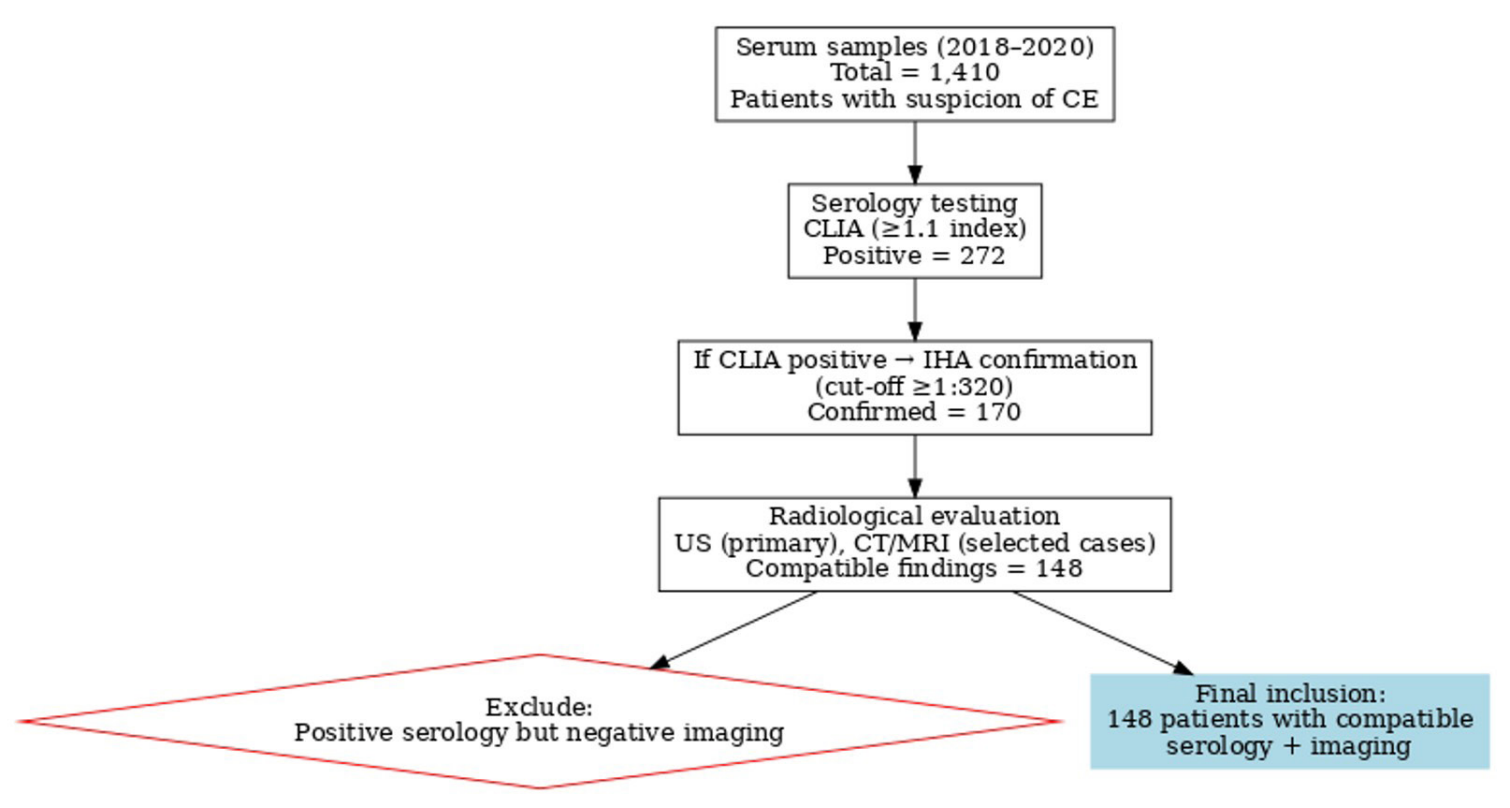
Diagnostic flow chart of the study population with number of cases at each step.

A flowchart was prepared to illustrate the diagnostic pathway followed in this study, starting with serological screening (CLIA and IHA), followed by radiological evaluation (US, CT, or MRI), and concluding with case inclusion for analysis. Consistently, serological testing was performed first, and radiological imaging was subsequently applied when indicated. The flowchart summarizes the stepwise approach applied in our institution and is presented in Figure 1.

## Results

A total of 1,410 serum samples were collected from 1,265 patients across various clinics for CE serology. Among these, 272 patients tested positive when both the CLIA and IHA methods were used, and 148 of them presented radiological findings compatible with CE. These 148 patients were the focus of our analysis, and the results are presented in Table 1. The majority of the patients (87.2%) were adults, with a mean age of 41.1 years (range 6–82), and female patients comprised 59.5% of the group. Nineteen patients were in the paediatric age group.

**Table 1:** Demographic and clinical characteristics of patients with cystic echinococcosis (Istanbul, Turkey, 2018–2020).

**Variable**	**Variable results**	**Regarding IgG CLIA** * **index values**	**P value**
**Number of the patients (n, %)**			
Total	148 (100%)	5.7 (1.1-11.7, 0.2)	0.09
Adult	129 (87.2%)	5.5 (1.1-11.7, 0.2)	
Pediatric	19 (12.8%)	6.7 (1.3-11.7, 0.7)	
**Gender (n, %)**			
Female	88 (59.5%)	5.3 (0.3)	0.06
Male	60 (40.5%)	6.2 (0.3)	
**Mean age (min-max, SE)**			
Total	41.1 (6-82, 1.5)		**0.003**
Female	45.0 (8-82, 2.0)		
Male	35.3 (6-72, 2.3)		
Pediatric	12.1 (6-17, 0.7)		
**Number of cysts, frequency (n, %)**			
Solitary	89 (60.1%)	5.2 (0.3)	**0.01**
Multiple	59 (39.9%)	6.4 (0.3)	
**Cyst diameter (n, %)**			
<5 cm	45 (30.4%)	5.1 (0.4)	0.20
5-10 cm	73 (49.3%)	5.7 (0.4)	
> 10 cm	30 (20.3%)	6.3 (0.4)	
**Cyst localization (n, %)**			
Only hepatic involvement	123 (83.1%)	5.51	0.08
Extrahepatic involvement	25 (16.9%)	6.62	
**Cyst stage (n, %)**			
Active (CE1, CE2, CE3a, CE3b)	95 (64.2%)	6.1	**0.01**
Inactive (CE4, CE5)	53 (35.8%)	4.9	

* CLIA: chemiluminescence immunoassay

### Radiological findings

US was the primary imaging method for 130 (87.8%) patients, whereas CT and/or MRI were employed for 18 (12.2%) patients. In our cohort, most abdominal cysts were diagnosed by US, whereas extrahepatic cysts, including pulmonary involvement, were confirmed using CT and MRI. Thus, pulmonary and other extrahepatic lesions were not diagnosed by US alone but required CT and/or MRI for confirmation. Single cysts were observed in 89 (60.1%) patients, whereas 59 (39.9%) had multiple cysts (p = 0.01). Multiple cysts were more prevalent in paediatric patients (63.2%) than in adult patients (36.4%) (p = 0.04). Extrahepatic cysts were identified in 14% of the adult patients and 36.8% of the paediatric patients (p = 0.01). The cyst diameter was smaller than 5 cm in 45 (30.4%) patients and larger than 10 cm in 30 (20.3%) patients. According to the WHO-IWGE classification, 64.2% of the cysts were categorized as active (CE1, CE2, CE3a, and CE3b), and 35.8% were categorized as inactive (CE4 and CE5). In 123 (83.1%) patients, the cyst was observed exclusively in the liver, and the lung was the second most affected organ in 17 (11.4%) patients (Table 2). The right lobe of the liver was the predominant location for cysts in 103 (69.6%) patients, whereas 21 (14.2%) patients presented with bilateral multiple liver cysts.

**Table 2:** Distribution of organ involvement among patients with cystic echinococcosis (Istanbul, Turkey, 2018–2020).

***Hepatic involvement***	**n**	**%**
Liver	123	83.1%
Liver + lung	14	9.4%
Liver + spleen	2	1.3%
Liver + ovary	1	0.7%
Liver + lung + spleen	1	0.7%
Liver + lung + spine	1	0.7%
Liver + spleen + bone + intraperitoneal	1	0.7%
***Extrahepatic involvement***		
Spleen	2	1.3%
Spleen + muscle + ovary	1	0.7%
Muscle	1	0.7%
Lung	1	0.7%
**Total**	**148**	**100%**

### Serological findings

The mean anti-*Echinococcus granulosus* IgG CLIA index was 5.7 (range 1.10–11.73) and was significantly higher in patients with active cysts than in those with inactive cysts (p = 0.01). Eosinophilia was observed in only three patients. No significant correlations were found between the IgG CLIA index and age or eosinophil count. The IgG CLIA index was higher in patients with multiple cysts than in those with solitary cysts, but this difference was not statistically significant. Cyst diameter was not significantly associated with age, the IgG CLIA index, or the eosinophil count. Additionally, the IgG CLIA index tended to be higher in patients with extrahepatic cysts, without reaching statistical significance (Table 1).

### Treatment and follow-up

Details of the patients' treatment and management procedures are outlined in Table 3. Among the patients, 47 (31.7%) underwent surgery, with surgery being the sole treatment for 12 (8.1%) patients. Percutaneous aspiration, injection of a scolicidal agent, and reaspiration (PAIR) were utilized in 25 (16.8%) patients. The watch-and-wait approach was adopted for 27 (18.2%) patients with uncomplicated inactive cysts. Relapse occurred in 12 patients, and rupture was observed in eight patients.

**Table 3:** Treatment and management of cystic echinococcosis cases (Istanbul, Turkey, 2018–2020).

**Treatment**	**n**	**%**
Watch-and-wait	27	18.2%
Chemotherapy	51	34.5%
Surgery and chemotherapy	33	22.3%
PAIR* plus chemotherapy	18	12.2%
Surgery	12	8.1%
PAIR	5	3.3%
Surgery and PAIR	1	0.7%
Surgery, chemotherapy and PAIR	1	0.7%
**Total**	**148**	**100%**

* PAIR: percutaneous aspiration, injection of a scolicidal agent, and reaspiration

## Discussion

Echinococcosis remains a significant public health concern in developing countries, particularly in rural areas, where risk is amplified by factors such as close contact with livestock, poor hygiene, and low educational levels (Kakamad et al., 2024). The HERACLES field survey, conducted in Romania, Bulgaria, and Turkey, revealed that among 24,693 individuals examined by US, abdominal CE was identified in 53 patients (0.59%) in Turkey (Tamarozzi et al., 2018). The primary modes of transmission involve contact with dogs and the consumption of contaminated water and food (Torgerson et al., 2020).

From a veterinary perspective, dogs and livestock play key roles in the life cycle of *E. granulosus*, acting as definitive and intermediate hosts, respectively. Insufficient control measures in rural areas allow for continuous transmission cycles, posing challenges for both the animal and human health sectors.

The prevalence of CE is reported to be greater in women (Budke et al., 2013), and in our study, women comprised 59.5% of the cases. However, there was no sex disparity concerning the number of cysts, location, size, complications, or treatment outcomes in seropositive patients.

In CE patients, the liver is the most commonly affected organ, with the right lobe being more commonly affected than the left lobe (Budke et al., 2013; Agudelo Higuita et al., 2016). The lung is the second most commonly affected organ (Agudelo Higuita et al., 2016; Wen et al., 2019). In our study, hepatic hydatid cysts were predominantly detected in the right lobe (103, 69.6%), often as single cysts (89, 60.1%). Extrahepatic cysts involving the lung, spleen, bones, abdominal cavity, muscles, and ovaries have been identified in 16.8% of cases (Pakala et al., 2016).

Multiple cysts were more prevalent in paediatric patients (63.2%) than in adults (36.4%) (p = 0.04). Extrahepatic cysts were found in 14% (n=18) of the adult patients and 36.8% (n=7) of the paediatric patients (p = 0.01). CE was detected at an earlier age in the presence of multiple cysts or cysts outside the liver.

Combining imaging techniques with serological tests has been suggested to enhance diagnostic performance in CE patients (McManus et al., 2012; Vola et al., 2019). Serological tests, such as IHA, ELISA, and IFA, are utilized in endemic regions, with IHA and ELISA being the preferred options (Agudelo Higuita et al., 2016; Pakala et al., 2016). Our study employed two first-line tests (CLIA and IHA) to ensure comprehensive serological diagnosis. Despite the lower specificity of IHA, its use in combination with CLIA reflects our institutional diagnostic protocol and provides complementary confirmation of results.

Among the investigated parameters, IgG CLIA index values were significantly associated with cyst activity (p = 0.01). Furthermore, the IgG CLIA index was higher in patients with extrahepatic and/or large cysts (Lissandrin et al., 2016). Recent studies have highlighted the importance of host immune responses, including IgG subclasses and cytokine profiles, in determining cyst activity and progression (Hamad et al., 2024). These findings support the role of immunological factors in complementing radiological staging for improved disease characterization.

In this study, we did not perform a direct quantitative comparison among serological and imaging modalities in terms of diagnostic performance. Imaging methods, particularly ultrasonography, were used as the confirmatory approach, while serological tests (CLIA and IHA) provided supportive evidence of infection. Therefore, the diagnostic relationship among CLIA, IHA, and imaging was interpreted qualitatively rather than statistically quantified. This limitation has been acknowledged, and future studies with larger datasets are warranted to compare diagnostic accuracy using ROC or logistic regression analyses.

While leukopenia, thrombocytopenia, and eosinophilia may be detected in some cases, laboratory tests for these conditions are generally not diagnostic for hydatid cyst disease (Botezatu et al., 2018). Eosinophilia was observed in only three patients in our study, potentially due to the absence of rupture and cyst leakage at the time of diagnosis.

These considerations are consistent with international standards. According to the latest WHO guidelines, serological tests are no longer recommended as stand-alone diagnostic tools, and imaging-based classification remains the gold standard for CE diagnosis (WHO, 2025).

Diagnostic tools such as US, radiography, CT, and MRI are commonly used, with US serving as the primary tool for abdominal CE, with a sensitivity range of 93–98% (Mandal & Mandal, 2012). Treatment options include surgery, percutaneous procedures, drug treatment, and the watch-and-wait approach (Brunetti et al., 2010). In our study, surgery was performed on 31.7% of the patients, while 16.8% underwent percutaneous procedures.

Control strategies include targeting stray dog populations, routine deworming, and public awareness programs for farmers and veterinarians, and these strategies are essential for interrupting the parasite’s transmission cycle and reducing the burden of CE in both humans and animals.

Additionally, the implementation of routine surveillance programs in livestock—particularly at slaughterhouses—can aid in the early detection and estimation of the CE burden in endemic regions. Strengthening intersectoral collaboration between veterinary authorities, farmers, and public health officials through structured deworming protocols in dogs and livestock tracing systems will enhance disease control under a One Health framework.

Our study has some limitations. As discussed above, this study primarily used descriptive statistics; diagnostic performance parameters such as sensitivity, specificity, and ROC analysis were not calculated. This limitation should be considered when interpreting the diagnostic power of the methods. The data were collected between 2018 and 2020 and may not fully reflect the most recent epidemiological trends of CE in Turkey. In addition, as this was a hospital-based retrospective analysis, the findings cannot be generalized to the wider population. Finally, because only patients with clinical suspicion of CE were included, the study does not represent a population-based screening and may be subject to selection bias.

## Conclusion

Although diagnostic tools have advanced, the detection of early-stage cysts remains challenging, as no single serological assay provides definitive confirmation. Therefore, novel serodiagnostic tests with broad applicability, high sensitivity, and improved specificity are needed. Our study highlights the importance of imaging combined with serology for the diagnosis and management of CE, underscoring integrated One Health approaches involving medical and veterinary sectors. The incorporation of veterinary surveillance data and the promotion of intersectoral collaborations are essential for effective CE control in endemic regions. These efforts align with the One Health framework and support comprehensive disease monitoring and prevention strategies.

## Data Availability

Aggregate (summary-level) data underlying the results and the analysis code are available from the corresponding author upon reasonable request. Individual-level data cannot be shared publicly due to patient privacy restrictions and institutional policy.
